# HSV1-induced enhancement of productive HIV-1 replication is associated with interferon pathway downregulation in human macrophages

**DOI:** 10.1590/0074-02760240102

**Published:** 2024-10-28

**Authors:** Viviane M Andrade, Filipe Pereira-Dutra, Juliana L Abrantes, Milene D Miranda, Thiago Moreno L Souza

**Affiliations:** 1 Fundação Oswaldo Cruz-Fiocruz, Instituto Oswaldo Cruz, Laboratório de Imunofarmacologia, Rio de Janeiro, RJ, Brasil; 2 Fundação Oswaldo Cruz-Fiocruz, Instituto Nacional de Ciência e Tecnologia de Inovação em Doenças de Populações Negligenciadas, Centro de Desenvolvimento Tecnológico em Saúde, Rio de Janeiro, RJ, Brasil; 3 Universidade Federal do Rio de Janeiro, Instituto de Ciências Biomédicas, Rio de Janeiro, RJ, Brasil; 4 Fundação Oswaldo Cruz-Fiocruz, Instituto Oswaldo Cruz, Laboratório de Morfologia e Morfogênese Viral, Rio de Janeiro, RJ, Brasil

**Keywords:** HHV1, coinfection, IFITM3, HIV-1 restriction factors

## Abstract

**BACKGROUND:**

Herpesviruses are common co-pathogens in individuals infected with human immunodeficiency virus (HIV). Herpes simplex virus type 1 (HSV1) enhances HIV-1 replication and has evolved mechanisms to evade or disrupt host innate immune responses, including interference with interferon (IFN) signalling pathways.

**OBJECTIVES:**

The aimed of this work was evaluated whether it HSV1 affects HIV-1 replication through the modulation of the IFN pathway in human macrophages.

**METHODS:**

Co-infections with HSV1 and HIV-1 were performed in monocyte-derived human macrophages (hMDMs). The production of infectious HIV-1 and HSV-1 was monitored 48 h post-coinfection. Additionally, mRNA and protein expression levels of interferon-stimulated genes (ISGs) were evaluated in both HIV-1-HSV1 coinfections and HSV1 mono-infections.

**FINDINGS:**

The HSV1 coinfection increasing the HIV-1 productive replication, following of downregulation of interferon-alpha (IFN-α) and interferon-induced transmembrane protein 3 (IFITM3) expression in hMDMs. Acyclovir treatment, in a dose-dependent manner, mitigated HSV1’s ability to decrease IFITM3 levels. Knockdown of HSV1 Us11 and virion host shutoff (VHS) genes reactivated the IFN pathway, evidenced by restored IFITM3 expression and activation of eIF2-α and PKR. This knockdown also returned HIV-1 replication to baseline levels.

**MAIN CONCLUSIONS:**

Our data suggested that HSV1 increases HIV-1 replication in human macrophages is associated with the downregulating interferon pathways and ISGs expression.

Human immunodeficiency virus (HIV) continues to be a major global public health issue, estimated that over 38 million people living with HIV currently in the world.^1^ ^,^ ^2^ The most advanced and late stage of HIV infection is acquired immunodeficiency syndrome (AIDS), marked by declining CD4^+^T lymphocytes numbers, increasing viremia levels, and greater susceptibility to potentially lethal opportunistic infections.^3^ ^,^ ^4^ To prevent the progression of the HIV infection to AIDS, the use of combined antiretroviral therapy (ART) is critical to effectively suppress the viral load, preserving the immune function and reducing the mortality of HIV^+^ patients.^2^ ^,^ ^3^

Co-infections and persistent immune activation contribute to HIV-associated pathogenesis, often increasing the viral load and viral persistence in HIV-infected people, even in those who use ART.^4^ ^,^ ^5^ ^,^ ^6^ Since the first reported cases of AIDS, coinfections with human alphaherpesviruses or herpes simplex viruses (HHV or HSV) are among the most common and well-demonstrated coinfections in HIV- positive people.^7^ ^,^ ^8^ In this context, several pieces of evidence show that HSV type 1 (HSV1, also known as HHV1) or type 2 (HSV2, also known as HHV2) coinfection enhances productive HIV infection *in vitro* ^9^ ^,^ ^10^ ^,^ ^11^ ^,^ ^12^ and increase HIV viral load in the plasma from patients.^13^ ^,^ ^14^ Despite HSV1/HHV1 being one of the most ubiquitous pathogens in the human population,^15^ ^,^ ^16^ most co-infection studies have focused on HIV-1 and HSV2/HHV2 interaction.^11^ ^,^ ^12^ ^,^ ^14^

During virus infection, the host’s immune response has a significant role in the control of virus infection.^17^ In this process, the interferons (IFN) signalling has a protagonist in the antiviral response, controlling hundreds of interferon-stimulated genes (ISGs) expression, including HIV-1 restriction factors.^17^ ^,^ ^18^ However, several viruses also evolved several strategies to subvert or inhibit IFN signalling.^18^ ^,^ ^19^ In this context, both HSV1 and HSV2 have evolved several strategies to suppress or subvert host innate immune signalling pathways, including impaired ISGs and restriction factors expression.(^19,20)^ Previous studies have demonstrated that several HSV1 late-stage replication proteins, such as ICP34.5, Us3, Us11, and virion host shutoff (VHS), impair IFN production or signalling by a still incompletely understood mechanism.^21^ ^,^ ^22^ ^,^ ^23^ ^,^ ^24^ Recent data show that HSV2 downmodulated IFN pathways and the expression of HIV-1 restriction factors, creating a microenvironment that augmented productive HIV infection in dendritic cells.^12^ However, the involvement of ISGs in the context of HSV1 and HIV coinfection is still unknown.

Based on the above, we hypothesised whether increased HIV-1 replication induced by co-infection with HSV1 is also associated with HSV1-interference in interferon pathways in human macrophages. Our data suggested that HSV1 increases HIV-1 replication in human macrophages is associated with the downregulating interferon pathways and ISGs expression.

## MATERIALS AND METHODS

*Statement of ethics* - Experimental procedures involving peripheral blood mononuclear cells (PBMCs) from buffy coat from healthy donors were performed with samples obtained after written informed consent and were approved by the Institutional Review Board (IRB) of the Oswaldo Cruz Foundation/Fiocruz (Rio de Janeiro, RJ, Brazil) under the number 397-07. The donors were HIV-negative and showed no clinical manifestations of HSV1.

*Cells, virus strain, and growth conditions* - PBMCs from healthy donors were obtained by density gradient centrifugation (Ficoll-Paque Premium; Cytiva, USA) from buffy coat preparations. Human monocyte-derived macrophages (HMDM) were obtained from PBMCs, through adherence to plastic plates, as described by Ferreira et al.^25^ Briefly, after density gradient centrifugation from buffy coats, PBMCs were plated onto 96-well plates in DMEM low-glucose containing 10% normal human serum (HS, Cytiva, USA) and penicillin-streptomycin (Sigma-Aldrich, USA) during six-seven days for monocyte differentiation into macrophages. Next, non-adherent cells were removed, and the macrophages were maintained in standard conditions in DMEM supplemented with 5% HS. Macrophage purity was above > 95%, as determined by flow cytometry analysis using anti-CD3 (BD Bioscience) and anti-CD68 (BD Bioscience) monoclonal antibodies.

African green monkey kidney cells (VERO cells, ATCC) and TZM-bl cells (obtained through the AIDS Research and Reference Reagent Program, Division of AIDS, National Institute of Allergy and Infectious Diseases; NIH, Bethesda, MD) were cultured in DMEM low-glucose supplemented with 10% heat-inactivated Hyclone Foetal Bovine Serum (FBS; Cytiva, USA), 100 U/mL penicillin, and 100 mg/mL streptomycin (Sigma-Aldrich, USA) at 37ºC in a 5% CO_2_ atmosphere.

HIV-1, monocytotropic, CCR5-dependent isolate HIV-1Ba-L was donated by the NIH AIDS Research and Reference Reagent Program (Division of AIDS, NIAID, NIH, Bethesda, MD). The HIV was expanded in phytohemagglutinin (PHA)-activated PBMCs from healthy donors, as described previously.^26^ HIV-1 viral titres were evaluated in cell-culture supernatants using HIV Type 1 P24 ELISA Kit (Cat# 0801111; ZeptoMetrix, USA), according to the manufacturer’s instructions. The viral stocks were aliquoted and stored at -70ºC for further studies.

HSV1 (KOS strain) was grown and titrated in Vero cells, as described previously by Miranda et al.^27^ Viral titres were determined by tissue culture infectious dose (TCID_50_/mL). The viral stocks were aliquoted and stored at -70ºC for further studies.

*HSV1 in vitro infection* - Human macrophages were plated (5 x 10^5^ cells/well) in 24-well culture plates (flat-bottom, tissue-culture-treated plates; Costar) and incubated for 12 h at 37ºC and 5% CO_2_. The cultures were then infected with HSV1 (MOI of 1.0) for 1 h at 37ºC. Noninternalised viruses were then removed by washing, and cell monolayers were replenished with fresh medium. In parallel, macrophage cultures were also stimulated with 10 ng/mL recombinant human IFN-2α (Cat# 10984-IF, R&D System). After this incubation period, cell monolayers were harvested for quantitative polymerase chain reaction (qPCR) and western blot analysis. To impair HSV1 infection, acyclovir (ACV; Cat# A0220000, Merk) was added to the cell culture and remained for all infection times at 37ºC in 5% CO_2_.

*HIV-1 in vitro infection and luciferase-based fluorescence viral titration assay* - Macrophages were infected with HIV-1 by exposing them overnight in viral suspensions containing 5-10 ng/mL of p24 antigen. Noninternalised viruses were then removed by washing, and cell monolayers were replenished with fresh medium. After 14 dpi, the cell monolayers were harvested for qPCR and western blot analysis. HIV-1 replication was evaluated in cell-culture supernatants by luciferase-based assay in TZM-bl, obtained from the NIH AIDS Reagent Program.^28^ Briefly, TMZ-bl cells were seeded onto a 96-well plate at 10^4^ cells/well. Cells were infected for 24 h. The quantification of the chemiluminescence signal was measured using Luciferase Assay System (Cat# E1500, Promega), according to the manufacturer’s protocol. The chemiluminescent signal was read in a LUMIstar Omega automated plate reader (BMG Labtech, Germany).

*HIV-1 - HSV1 coinfection* - HIV-1-infected macrophages (12 days of infection) were inoculated with HSV1 (MOIs of 0,1 or 1) for 1 h at 37ºC. Noninternalised viruses were then removed by washing, and cell monolayers were replenished with fresh medium. After 48 h post coinfection, cell monolayers were harvested for qPCR and western blot analysis, and the supernatants were harvested for quantification of viral titres.

*RNA extraction, cDNA synthesis, real-time qPCR* - Total RNA was extracted from macrophages seeded in 24-well plates (10^5^ cells/well) using Qiagen RNeasy Mini Kit (Qiagen, Germany), following the manufacturer’s protocol. The concentration of total RNA was determined using Qubit Quantitation Fluorometer and Quant-iT reagents (ThermoFisher Scientific, USA). The first strand of cDNA was synthesised from 1 μg of total RNA using RT^2^ First-Strand Kit (Qiagen, USA) or MMLV Reverse Transcriptase (ThermoFisher Scientific, USA), and 300ng of Random Hexamer primes (ThermoFisher Scientific, USA) as the anchor primer.^29^

For the qPCR reaction, 1.5 μL of template cDNA (approximately 50 ng) was mixed with 300nM of each primer (Table I), and 1x SYBR^®^ Green qPCR master mix (Applied Biosystems, USA) Alternatively, the mRNA levels for the RFs were evaluated by the RT2 Profiler PCR Array (CAPH12347A) (Qiagen, Germany), composed of primers for different IFN-induced restriction factors, such as *APOBEC3G* (*Apolipoprotein B MRNA Editing Enzyme Catalytic Subunit 3G*, cat # PPH06904A), *IFITM1* (*Interferon Induced Transmembrane Protein* 1, cat # PPH05981C), *IFITM2* (*Interferon Induced Transmembrane Protein 2*, cat # PPH05548F), *IFITM3* (*Interferon Induced Transmembrane Protein 3*, cat # PPH02872E), *IFN1-α* (*Interferon 1-α*, cat# PPH01321B), *IFNR* (*Interferon Receptor*, PPH00869F), *MX1* (*Myxovirus Resistance Protein 1*, cat # PPH01325A), *MX2* (*Myxovirus Resistance Protein 2,* cat # PPH01326F), *MCPIP1* (*Monocyte Chemotactic Protein-Induced Protein 1*, cat # PPH16134B), *SAMHD1* (*SAM domain and HD domain-containing protein 1*, cat # PPH18140A), *TETHERIN / BST-2* (cat # PPH05790B) and the housekeeping gene *GAPDH* (*Glyceraldehyde 3*-*phosphate dehydrogenase,* cat# PPH00150F). The qPCR reactions were performed using a 7500 Fast Real-Time PCR system (Applied Biosystems). The relative mRNA expression was calculated by the 2^− ΔΔCt^ method.

**TABLE I:** Primes sequences for amplification of herpes simplex virus type 1 (HSV-1) genes

Gene symbol	Gene description	Genome position (NC_001806.2)	Primers (F: forward, R: reverse)	Ta (°C)	Amplicon size
Us3	Serine/threonine protein kinase US3	135328 - 135470	F 5’- CCTTTTATACCCCAGCCGAG -3’ R 5’- TGCCTGTCAAACTCTACCAC-3’	60 ± 1	143 bp
RL1	Neurovirulence protein ICP34.5	125773 - 125902	F 5’- TGCCTGTCAAACTCTACCAC -3’ R 5’- GTTACCTGGGACTGTGCG -3’	60 ± 1	130 bp
Us11	Envelope glycoprotein I	145066 - 145215	F 5’- GGCGACCCAGATGTTTACTTA -3’ R 5’- ACCCGAATCTCCACATTGC -3’	60 ± 1	150 bp
VHS	Virion host shut off	91833 - 91981	F 5’- TGGGCTGTGATATTGTGTTGG -3’ R 5’- GTAGGTGTTATTGGGATGGAGG -3’	60 ± 1	149 bp

*Western blot assay* - Cellular extracts of 1x10^6^ cells were homogenised in RIPA lysis buffer (1% Triton X-100, 2% SDS, 150 mM NaCl, 10 mM HEPES, 2 mM EDTA, pH 8.0) containing protease and phosphatase inhibitor cocktail (Roche, Switzerland). After centrifugation at 13 000 × g for 5 min, cell lysates were prepared under reducing and denaturing conditions and subjected to sodium dodecyl sulphate-polyacrylamide gel electrophoresis (SDS-PAGE). Protein aliquots of 20 μg of the cell lysates were fractionated by electrophoresis on 10% acrylamide gels. The proteins were transferred onto a nitrocellulose membrane (Cytiva, USA), followed by the blocking of nonspecific binding sites in 5% non-fat milk in TBST (50 mM Tris-HCl - pH 7.4, 150 mM NaCl, 0.05% Tween 20) for 1 h at room temperature and blotted with primary antibodies in TBST overnight at 4ºC. The following antibodies were used: anti-IFITM3/Fragilis (EPR26405-14) (Abcam, Cat# ab288563), anti-APOBEC3G (Abcam, Cat# ab194581), anti-BST-2/Tetherin (EPR20202-150) (Abcam, Cat# ab243230), anti-PKR (Cell signalling, Cat#3072), anti-phospho-PKR (Thr451) (Millipore/Merck, Cat# 07-886), anti-eIF2a (Cell signalling, Cat#9722), anti-phospho-eIF2a (Ser51) (Cell signalling, Cat#9721), and anti-α-tubulin antibodies (Sigma, Cat# T5168). Proteins of interest were identified by incubating the membrane with HRP-conjugated secondary antibodies in TBST, followed by detections by Supersignal Chemiluminescence (Cytiva, USA).

*Measurements of IFN-α and IFN-β* - IFN-α and IFN-β in cell-free culture supernatants were measured using mouse Duoset ELISA kit (R&D Systems, USA) according to manufacturer’s instructions.

*HSV1 knocking down assays* - Human macrophages were plated (2 x 10^6^ cells/well) in 12-well culture plates (flat-bottom, tissue-culture-treated plates; Costar) and incubated for 12 h at 37ºC and 5% CO2. Cells were transfected with 10 pmol/μL siRNA targeting HSV1 genes US3, ICP34.5, US11, and VHS or scramble sequence (SCR) (Table II) (ThermoFish Scientific, USA) in Opti-MEM (Gibco, USA), using Lipofectamine 2000 (Sigma-Aldrich, USA). Alternatively, eleven-day-old HIV-1-infected macrophages were also transfected with siRNAs for VHS and US3 genes. After 24 h of recovery, HMDMs were then infected with HSV1. To evaluate the efficiency of transfection in macrophages, we transfected macrophages varying both concentrations of Silencer^®^ FAM™-Labelled GAPDH siRNA (Promega) and lipofectamine. The efficiency of transfection was measured 6-24 h post-transfection through green filter fluorescence, showing a mean delivery efficiency above or equal to 90%.

**TABLE II:** siRNA sequences for knockdown assay

Targets	siRNA sequences
HSV1 US3	5’-CCGTATACCACGACCGTCGACATTT-3’
HSV1RL1	5’-GCCCACTTCCCGGTATGGTAATTAA-3’
HSV1 US11	5’-CACTACGATCTCGAAGCCATCTGAA-3’
HSV1 VHS	5’-GACATGGGTTTGTTCGGGATGATGA-3’
Scramble 1	5’-CACTAGCGCTCCGAACTACTATGAA-3’
Scramble 2	5’-GACTGGGTGTTGGCTGTAGATATGA-3’

*Statistical analysis* - All experiments were carried out at least three times independently, including technical replicates in each assay. Statistical analysis was carried out using GraphPad Prism software. Student’s T-test was used to compare differences between the two groups. One-way analysis of variance (ANOVA) was used to compare differences among three groups following a normal (parametric) distribution with Tukey’s post-hoc test was used to determine significant differences between groups; p values < 0.05 were considered statistically significant.

## RESULTS

**Fig. 1:**
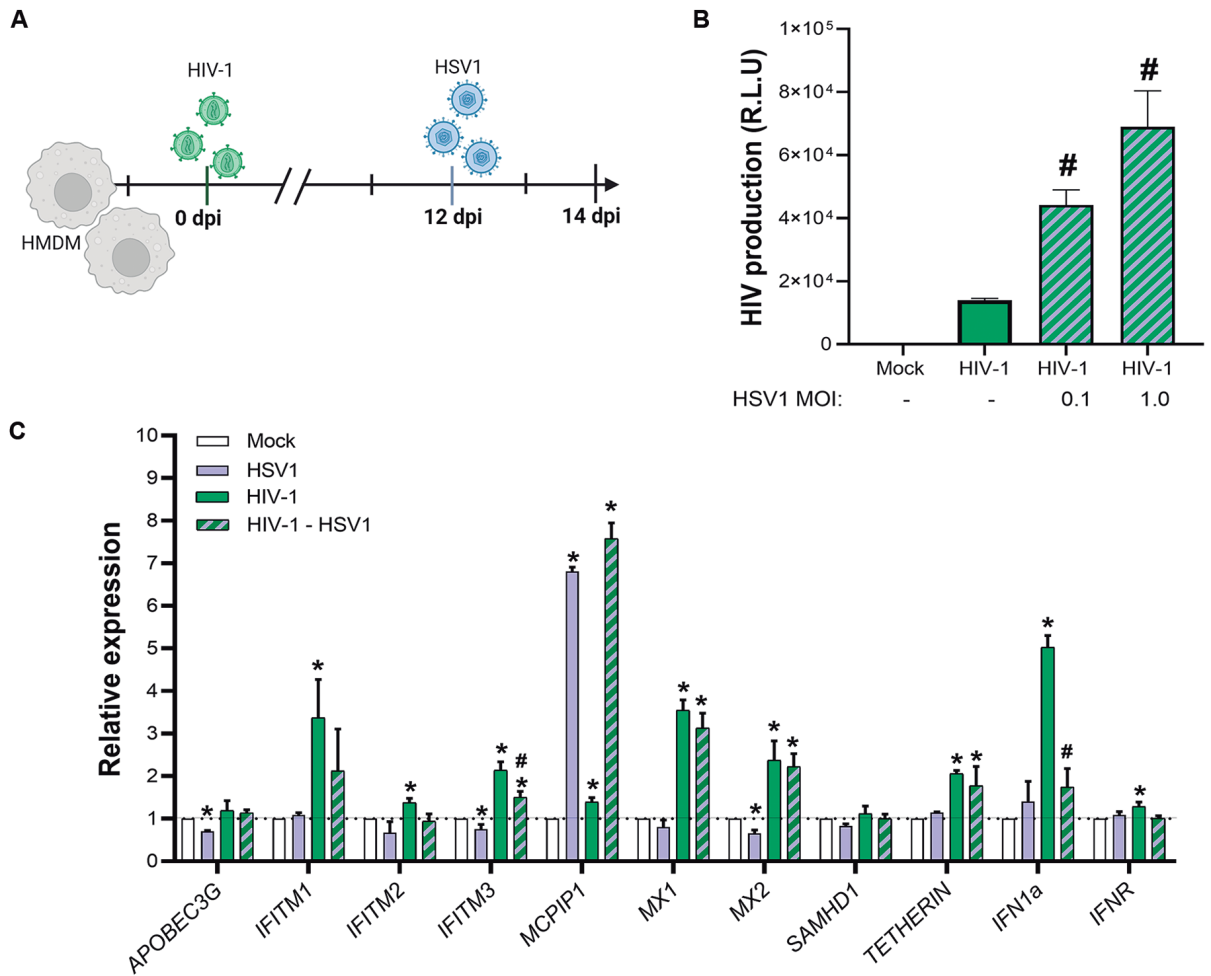
herpes simplex virus type 1 (HSV-1) coinfection increasing human immunodeficiency virus (HIV)-1 proliferation and impaired the expression of IFITM3, and IFN-1 induced by HIV infection in human macrophages. (A) Human macrophages (hMDMs) were infected with HIV-1 for 12 days and co-infected with HSV1 at the indicated multiplicity of infection (MOI). After two days of coinfection, the supernatants were harvested, and the virus titre was measured. (The image was created with Biorender.com). (B) HIV-1 titre through luciferase-based assay in TZM-bl cells. Bars are the mean ± standard error of the mean (SEM); n = 3. (C) Relative restriction factors mRNA in macrophages measured by quantitative polymerase chain reaction (qPCR) analysis. Data were normalised by *Glyceraldehyde 3-phosphate dehydrogenase* (*GAPDH*) control. Bars are the mean 2^−ΔΔCt^± SEM; n = 3. One-way ANOVA with Tukey’s *post-hoc* test. *p < 0.05 in comparison to mock; #p < 0.05 comparison to HIV-1 mono-infected macrophages. APOBEC3G: apolipoprotein B MRNA editing enzyme catalytic subunit 3G; IFITM1: interferon induced transmembrane protein 1; IFITM2: interferon induced transmembrane protein 2; IFITM3: interferon induced transmembrane protein 3; IFN1-α: interferon 1-α; IFNR: interferon receptor; MX1: myxovirus resistance protein 1; MX2: myxovirus resistance protein 2; MCPIP1: Monocyte Chemotactic Protein-Induced Protein 1, SAMHD1: SAM domain and HD domain-containing protein 1.

*HIV-1 - HSV1 coinfection increases HIV-1 replication, but not of HSV1, in human macrophages* - Human macrophages can be infected with HIV-1 and HSV1.^30^ However, HIV-1 has a slower replication rate in macrophages,^31^ with a replicative peak at 14 days post-infection (dpi).^26^ On the other hand, the pick of replication of HSV1 is 48 h post-infection (hpi).^16^ We first investigated whether a subsequent infection by HSV1 could modulate HIV replication in macrophages. To synchronise the peaks of HSV1 and HIV-1 replication, which occur around 48 h and 14 days, respectively, we infected 11- to 12-day-old HIV-1-infected macrophages with HSV1. We then monitored the production of infectious HIV-1 and HSV1 48 h post-coinfection (Fig. 1A). The infection HIV-1 particles were quantified by luciferase-based assay in TMZ-bl assay, obtained from the NIH AIDS Reagent Program. Luciferase activity was quantified by luminescence and is directly proportional to the number of infectious virus particles present in the initial inoculum.^28^ In this assay, we observed that HSV1 coinfection augments infective HIV-1 production in human macrophages after 48 h post-coinfection when compared to HIV mono-infection (Fig. 1B).

Moreover, we have found that HSV1 subsequently infection induced increasing HIV-1 replication through the phenomenon MOI-dependent manner (Fig. 1B). At the MOIs of 0.1 and 1 of HSV1, production of HIV-1 was 3 and 7-fold increase, respectively, when compared to the basal retroviral production. Of note, we also measured HSV1 titres in macrophages during coinfection. By titrating HSV1 in Vero cells, we observed that the HSV1 replication in macrophages was marginal (10^2^ e 10^3^ TCID/mL at 0.1 and 1 MOIs) and not affected by the presence of HIV-1 (10^2^ e 10^3^ TCID/mL at 0.1 and 1 MOIs).

*HSV1 impaired the expression of IFITM3 and IFN1a gene expression in HIV-infected macrophages* - Restriction factors (RFs) expression is critical for the control of HIV replication in macrophages.^8^ ^,^ ^32^ To evaluate if an HSV1-induced increase of HIV-1 replication could be associated with impaired of RFs expression, we evaluated the expression of 10 RFs genes key in HIV infection. As previously reported, we observed that HSV1 and HIV-1 infection have different dynamics in the induction of RFs.^33^ ^,^ ^34^ We found that HSV1 down-regulates the basal levels of *APOBEC3G*, *IFITM3,* and *MX2*, and up-regulated the expression of the *MCPIP1* gene (Fig. 1C). On the other hand, HIV-1 infection triggers the over-expression of several RFs genes, including *IFITM1*, *IFITM2*, *IFITM3*, *MX1*, *MX2*, *TETHERIN*, *IFN1a,* and *IFNR* (Fig. 1C). In the coinfection, HSV1 was able to decrease the HIV-1-related augment of *IFITM3* and *IFN1a* (Fig. 1C).

**Fig. 2:**
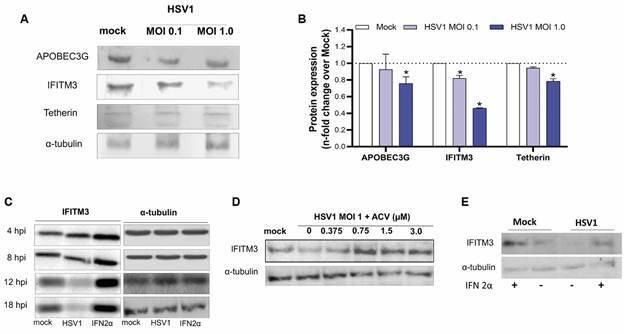
IFITM3 protein expression are reduced during late events of herpes simplex virus type 1 (HSV-1) infection. Human macrophages (hMDMs) were infected with HSV1 at the indicated multiplicity of infection (MOI). After two days of coinfection, the supernatants were harvested, and the cells were lysed. (A) Cell lysates were collected for the detection of APOBEC3G, IFITM3, and Tetherin by Western blotting. α-tubulin levels were used for control of protein loading. (B) Western blotting densitometry of APOBEC3G, IFITM3, and Tetherin after loading normalisation with α-tubulin. The images were analysed with ImageJ software version 2.01. Bars are the mean 2^−ΔΔCt^± standard error of the mean (SEM); n = 3. One-way ANOVA with Tukey’s *post-hoc* test. *p < 0.05 in comparison to mock; #p < 0.05 comparison to human immunodeficiency virus (HIV)-1 mono-infected macrophages. (C) hMDM were either infected with MOI 1 of HSV1 or treated with interferon 2-apha (IFN2α) (10 ng/mL) for 24 h. Monolayers were lysed using at the indicated time-points for analysis of IFITM3 expression and α-tubulin, as a housekeeping control; (D) Cells were infected with HSV1 at MOI 1, followed by treatment with increased concentrations of acyclovir (ACV). After 24 h incubation cells were lysed and submitted to blotting assays for IFITM3 protein expression and α-tubulin. All Figures are representative of at least three independent replicates. APOBEC3G: apolipoprotein B MRNA editing enzyme catalytic subunit 3G; IFITM3: interferon induced transmembrane protein 3; *GAPDH*: *Glyceraldehyde 3-phosphate dehydrogenase*.

*The reduction of IFITM3 protein levels is a late event during HSV1 infection in human macrophages* - Our next step was to evaluate if HSV1-induced down-modulation of RFs mRNA also affects the RFs protein levels in the macrophages. We observed that HSV1 MOI 1.0 infection was able to reduce the APOBECG, Tetherin, and IFITM3 expression in macrophages (Fig. 2A-B). However, only IFITM3 has a decrease in protein expression in an MOI-dependent manner (Fig. 2A-B). Next, we investigated whether the downregulation of IFITM3 could be an early or late event during HSV1 infection. We observed a reduction in IFITM3 levels beginning at 8 hpi (Fig. 2C) and declining thereafter. This time frame corresponds to the beginning of the late stages of HSV1 replication.^16^ On the other hand, IFITM3 expression increased in the presence of IFN-α, demonstrating that this protein is an ISG (Fig. 2C). Since late HSV1 proteins are synthesised after viral DNA replication,^35^ blockage of HSV1 DNA polymerase would prevent the HSV1-induced down-modulation of IFITM3. To pharmacologically confirm this insight, we analysed IFITM3 levels in HSV1-infected macrophages treated with acyclovir (ACV). The treatment with ACV limited HSV1’s ability to reduce IFITM3 in a dose-dependent manner (Fig. 2D). Late events that occur after HSV1 DNA replication may be triggering the reduction in IFITM3 levels.

**Fig. 3:**
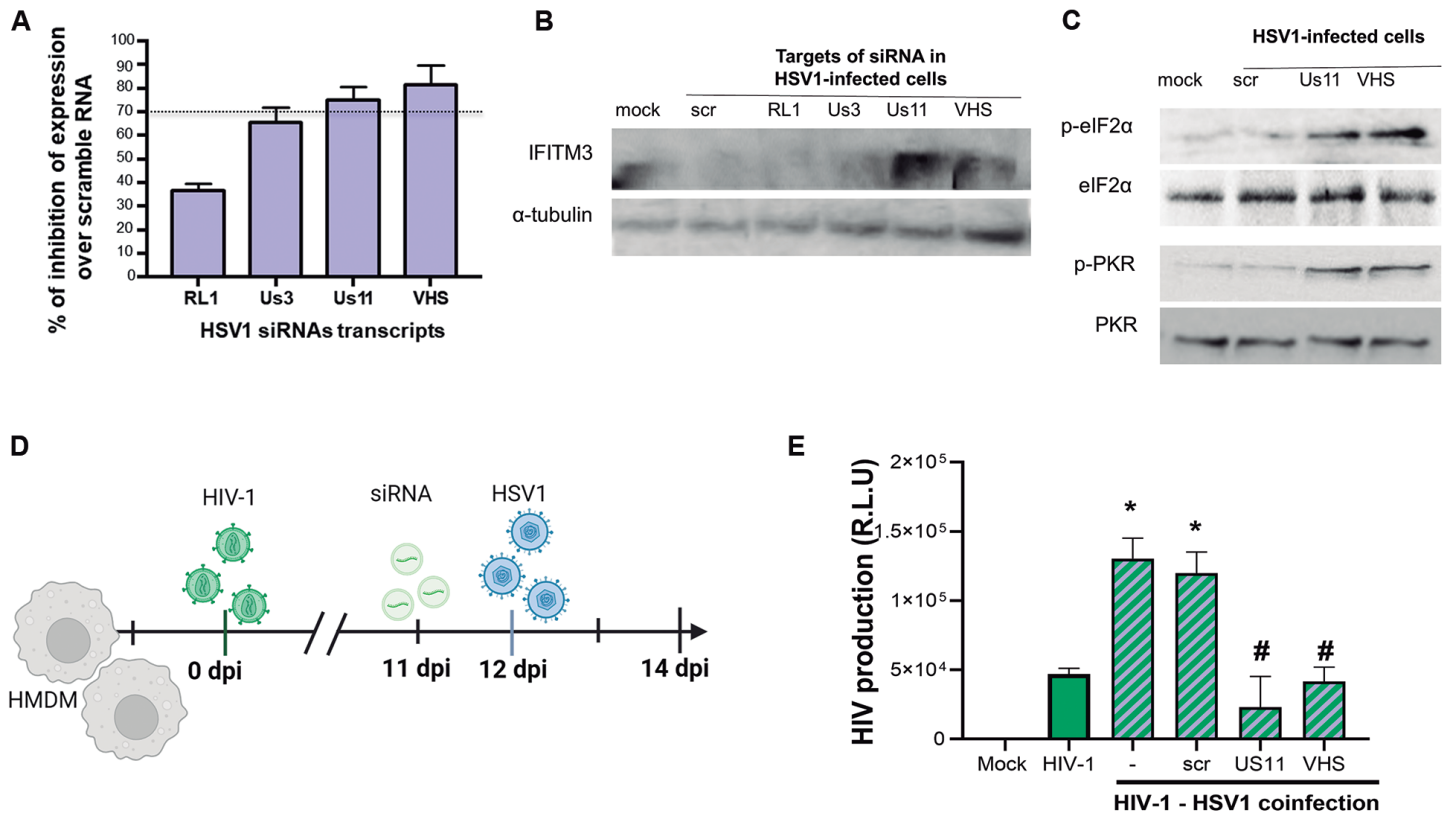
antiviral protein expression is rescued after silencing of herpes simplex virus type 1 (HSV-1) late phase genes. (A) Human macrophages (hMDM) were either infected with multiplicity of infection (MOI) of 1 of HSV1 and/or treated with interferon 2-alpha (IFN2α) (10 ng/mL) for 24 h. Monolayers were lysed using at the indicated time-points for analysis of IFITM3 expression and α-tubulin, as a housekeeping control; (B-D) hMDMs were transfected with 10 pmol of small interfering RNA (siRNA) for the following HSV1 transcripts: RL1, Us3, Us11 and virion host shutoff (VHS) and infected with HSV1 at 1 MOI. (B) After 24 h infection, monolayers were lysed and RNA extracted, followed by complementary DNA (cDNA) synthesis and quantitative polymerase chain reaction (qPCR) analysis of HSV1 viral genes. Bars are the mean ± standard error of the mean (SEM); n = 3. (C) IFITM3, (D) PKR and eIF2α total and phosphorylated protein content, were assessed by immunoblotting. (E-F) hMDMs were infected with human immunodeficiency virus (HIV)-1 for 12 days and then transfected with Us11 and VHS siRNAs. After 24 h post transfection cells were co-infected with HSV1. After a total of 14 days of HIV-1 infection, supernatants were harvested and the (F) HIV-1 levels was quantified by luciferase-based assay. Bars are the mean ± SEM; n = 3. One-way ANOVA with Tukey’s *post-hoc* test. *p < 0.05 in comparison to HIV-1 mono-infection, #p < 0.05 comparison to scramble siRNA co-infected macrophages. IFITM3: interferon induced transmembrane protein 3; PKR: dsRNA-activated protein kinase; eIF2-α: activation of eukaryotic initiation factor 2-α.

*Late-phase HSV1 proteins Us11 and VHS negatively-regulate IFITM3 content* - As the expression of IFITM3 is dependent on IFN signalling,^36^ we evaluated if type if type I IFN replacement could reverse the HSV1-induced decreasing IFITM3 expression. We indeed observed that the addition of IFN type I to the culture was able to restore IFITM3 levels reduced by HSV1 infection (Fig. 2E). Of note, in another set of experiments evaluated whether infection and co-infection affected the basal IFN-α or IFN-β levels in the macrophage culture supernatant, but all supernatants had values below the limit of detection for this protein. The challenge of quantifying IFN-α or IFN-β in macrophages infected with HIV has also been documented by others.^26^ ^,^ ^37^ ^,^ ^38^ To advance on the functional engagement of downstream pathways of IFN-α signalling, our next step was to monitor whether HSV1 late genes would be responsible for IFITM3 modulation. Previous studies have demonstrated that several late-stage replication proteins of HSV1, such as RL1/ICP34.5, Us3, Us11, and VHS, interfere with IFN signalling.^21^ ^,^ ^22^ ^,^ ^23^ ^,^ ^24^ Thus, we used siRNA to knockdown the synthesis of HSV1 genes *RL1, Us3, Us11*, and *VHS*. We verified that this strategy of knockdown was more efficient for HSV1 Us11 and VHS transcripts (72 and 78% of inhibition over infected control cells, respectively) than for Us3 and RL1 (65% and 40% of inhibition, respectively) (Fig. 3A). We subsequently measured the IFITM3 levels of HSV1-infected cells after transfection with all these siRNAs. We found that only *Us11* and *VHS* knockdown restored the levels of IFITM3 (Fig. 3B).

To confirm that HSV1 *Us11* and *VHS* effects on IFITM3 expression are related to the antagonism of the IFN signalling, we investigated the levels of double-stranded RNA-dependent protein kinase R (PKR) and eIF2-α phosphorylated and total were measured upon transfection with siRNAs. The PKR/eIF2-α system is among the earliest and canonical ISGs activated by the interferon pathway.^20^ ^,^ ^39^ Whereas HSV1 infection prevents the phosphorylation of PKR and eIF2-α to classically antagonise IFN signalling, knockdown of *Us11* and *VHS* restores this arm of the innate immune response (Fig. 3C). Our next step was to verify whether the knocking down of Us11 and VHS during HSV1 replication could also prevent this herpesvirus-induced enhancement of HIV-1 infection (Fig. 3D). We found that HSV1-induced enhancement of HIV-1 replication is preserved in scrambled siRNA controls and prevented when the late herpesvirus genes Us11 and VHS are targeted (Fig. 3E). Moreover, both Us11 and VHS knockdown limited HIV-1 replication to basal levels (Fig. 3E). Altogether, our results suggests that HSV1-induced enhancement of HIV-1 replication may be linked to interferon pathway dysregulation.

## DISCUSSION

Infection with HHV or HSV, known as herpes, is one of the most ancient and common in the world population.^8^ ^,^ ^11^ HSV1 establishes life-long persistent infections, alternating periods of latency, and reactivation under the vulnerability of the immune system.^40^ During the reactivation, HSV1 promotes immune activation, recruiting activated CD4^+^ T cells, macrophages, and dendritic cells to the skin/mucosa, resulting in the formation of ulcerative lesions.^19^ ^,^ ^40^ This inflammatory process has a central role in the clearance of HSV1 particles, fundamental to restricting HSV1 growth.^41^ On the other hand, HSV-induced inflammation creates an HIV-supportive milieu, because CD4^+^ T cells and macrophages are permissive to HIV infection.^6^ ^,^ ^26^ Moreover, macrophages are considered important HIV latent cellular reservoirs.^12^ ^,^ ^31^ In this context, we observed that HSV1 enhances the activation of HIV-1 replication in the macrophages, which can impose an additional challenge faced by HIV-infected individuals.

Various molecular mechanisms have been reported over the last three decades to be involved in HSV1-enhanced HIV-1 replication.^9^ ^,^ ^42^ ^,^ ^43^ ^,^ ^44^ One of the more important was the description of HSV1 protein Us11 can carry with newly synthesised viral particles and binds to the rev responsible elements (RRE) in the HIV-1 mRNA, which may be interfering directly in the formation of new HIV-1 particles.^42^ ^,^ ^43^ On the other hand, *HSV1 VHS* is an RNase that destabilises both cellular and viral mRNAs, suppressing host protein synthesis, and stimulating the translation of viral mRNAs.^45^ ^,^ ^46^

Due to prolonged coevolution with its host, HSV1 has developed multiple mechanisms to attenuate host antiviral defences and facilitate its infection.^47^ ^,^ ^48^ ^,^ ^49^ Like observed in HSV2 infection in dendritic cells,^12^ we also observed that HSV1 infection modulates the expression of IFN-stimulated restriction factors. In our model, HSV1 downregulated IFITM3, both the basal level as well induced by HIV-1 coinfection. On the other hand, IFN-α replacement reversed the HSV1-induced decrease in IFITM3. The IFITM3 is a small transmembrane RFs central to reducing HIV-1 infectivity and viral protein production by a still incompletely understood mechanism.^50^ ^,^ ^51^ ^,^ ^52^ A few pieces of evidence suggests that both overexpressed and endogenous IFITM proteins reduce HIV infectivity and spread by being incorporated into budding virions in producer cells.^53^ In this context, the downregulation of IFITM3 by HSV1 coinfection could enhance HIV infectivity, as evidenced by the increase in HIV1 productive replication.

Several HSV1genes suppressed the interferon signalling pathway, inhibited type I interferon production, and/or the activities of ISGs.^33^ ^,^ ^54^ ^,^ ^55^ In this context, the HSV1 US11 gene encodes an RNA-binding protein (RBP) that inhibits PKR phosphorylation. This RBP can bind viral dsRNA to prevent PKR recognition.^23^ ^,^ ^56^ The expression and activation of IFN-inducible PKR system is one of the earliest and classical response to infection induced by the type I IFN response.^22^ ^,^ ^39^ ^,^ ^57^ In addition to restoring IFITM3 expression, the knockdown of HSV1 Us11 and VHS also re-established the activation of eIF2-α and PKR, another distinct axis of the interferon pathway. Furthermore, all these proteins are important for controlling HIV replication.^37^ ^,^ ^52^ ^,^ ^58^ During virus infection, PKR is activated and phosphorylates eIF2α, which inhibits viral protein synthesis and plays a crucial role in antiviral innate immunity.^39^ ^,^ ^57^ At the onset of HIV infection, PKR is transiently activated, contributing to a block in viral expression and replication.^58^ The *HSV1 VHS* also dampens antiviral responses by suppressing PKR activation^59^ and IFN-β response.^22^ ^,^ ^45^ Moreover, Us11 and VHS knockdown also restored the HIV replication to basal levels in macrophages. In this context, when we restore the expression/activation of IFITM3, PKR, and eIF4e by silencing *HSV1 VHS* and *Us11,* and observe HIV-1 replication returning to basal levels during co-infection, it suggests that HSV1-induced enhancement of HIV-1 replication may be linked to interferon pathway dysregulation. Of note, it is difficult to rule out *Us3* and *RL1* contributions to the modulation of IFITM3 levels, because these transcripts were knockdown with lesser efficiency than Us11 and VHS.

As reported by Moriuchi et al.,^30^ the HSV1-induced augment of HIV replication does not appear to be dependent on productive HSV1 replication in macrophages. In fact, HSV1 replication in macrophages was marginal and unaffected by HIV-1 coinfection. However, we observed that even though HSV1 replication is not productive, it can modulate the host cell response. A similar result was also observed for HSV2 in the coinfection with HIV-1 in dendritic cells,^12^ regardless of whether HSV2 was the first or second infection in this model. These results suggest that even viruses that do not replicate productively may play a key role in the pathophysiology of coinfections of other viruses.

In summary, our findings propose the involvement of interferon pathways as a novel component in the intricate coinfection of HSV1 and HIV-1. The knockdown HSV1 late genes Us11 and VHS re-established the IFN pathway, which can be inferred through the rescue of IFITM3 expression and the activation of eIF2-α and PKR. Moreover, HSV1 Us11 and VHS knockdown restored the HIV-1 replication to basal levels in macrophages. Our findings motivate further translational studies in the pathogenesis of HIV-1-infected individuals since macrophages are important retrovirus reservoirs that could often co-host HSV1.
